# Supplementary figures and images for: Risk of HBV reactivation in HBV/HCV-co-infected HCV-treated patients: A single-center study
Source: PLoS One. 2025 May 30;20(5):e0324019. doi: 10.1371/journal.pone.0324019 (PMC12124557; doi:10.1371/journal.pone.0324019)

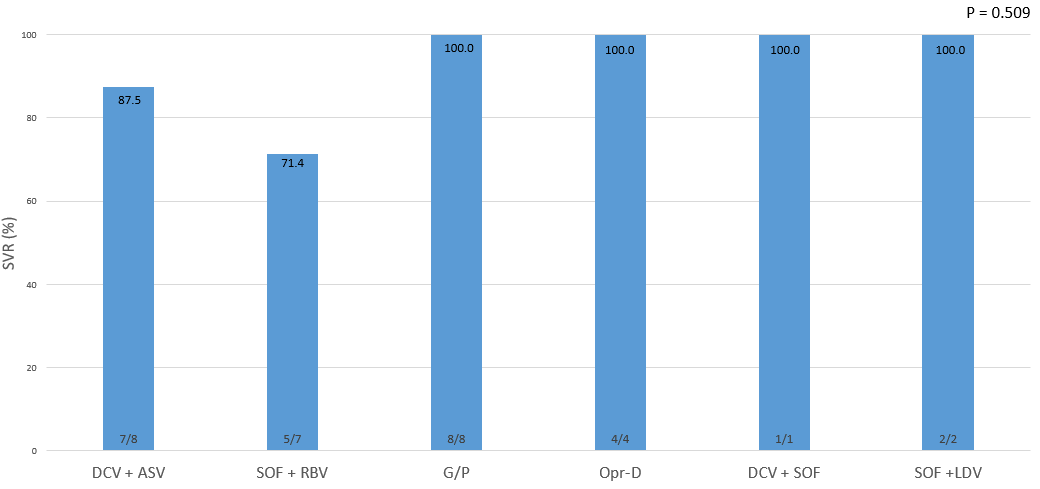

Supplement: S1 Fig — HBV, hepatitis B virus; HCV, hepatitis C virus; DAA, direct-acting antiviral; DCV, daclatasvir; ASV, asunaprevir; SOF, sofosbuvir; RBV, ribavirin; G/P, glecaprevir/pibrentasvir; OPr-D, ombitavir/paritaprevir/ritonavir plus dasabuvir; LDV, ledipasvir, SVR, sustained virological response. (TIF) [file pone.0324019.s001.tif]
