# Supplementary material for: Risk of HBV reactivation in HBV/HCV-co-infected HCV-treated patients: A single-center study
Source: PLoS One. 2025 May 30;20(5):e0324019. doi: 10.1371/journal.pone.0324019 (PMC12124557; doi:10.1371/journal.pone.0324019)
Supplement: Attachment — Submitted filename: Response to Reviewers_20250126_final (1).docx [file pone.0324019.s004.docx]

**Reviewer #1:** In the manuscript, the authors described the HBV reactivation in patients coinfected with HCV treated with different regimens. This issue is important in the field since worldwide HCV DAA regimens are largely employed and clinicians should be aware of this possibility.
- The manuscript requires Major Compulsory Revisions highlighted in my comments

TITLE

1. Risk of HBV Reactivation among HBV/HCV Coinfected Patients Treated with Direct-Acting Antiviral Agents: A Single-Center Experience
   The title and objectives of the abstract must be corrected. The authors analyzed other determinants of HBV reactivation not only in DAA but also in IFN-treated patients.
   Suggestion: Risk of HBV Reactivation among HBV/HCV Coinfected Treated Patients: A Single-Center Study
   → **Thank you for your suggestion. We have revised the title of the paper to “Risk of HBV Reactivation in HBV/HCV-Co-infected HCV-Treated Patients: A Single-Center Study” as per your suggestion. This title accurately reflects the subject and content of the paper.**

ABSTRACT

2. followed for a median follow-up of 53 months
   - correct: followed for a median of 53 months
   → **Thank you for your thoughtful comment. We have corrected the error and made the following changes: “This study focused on 47 patients with chronic hepatitis C (CHC) and positivity for HBV surface antigen (HBsAg) who were treated with interferon (IFN)-based therapy, DAA, or DAA after IFN-based therapy failure and followed for a median of 53 months.”**

3. Conclusion: The conclusion is not supported by the objectives, methodology and results. Furthermore, what does this study add to the field of HBV/HCV coinfected treated patients?

→ **Thank you for your comment. This study aimed to investigate the frequency and timing of HBV reactivation in patients with HBV/HCV co-infection undergoing different types of HCV treatment. Approximately 30% of patients experienced HBV reactivation after HCV therapy. The results indicate that HBV reactivation is more common and occurs earlier in patients receiving DAA treatment after IFN-based treatment failure. This study emphasizes the importance of testing all CHC patients for HBV exposure before DAA treatment and closely monitoring HBsAg-positive patients for HBV reactivation during DAA therapy. These findings contribute significantly to the understanding of the risk of HBV reactivation and provide practical guidelines for managing this risk in clinical practice, ultimately improving the care of HBV/HCV-co-infected patients.**

**Your input has been thoroughly considered, and the revised conclusion is as follows: “HBV reactivation in HBsAg-positive CHC patients is more common and occurs earlier in those who receive DAA treatment after IFN-based treatment failure than in those with IFN-based treatment. Therefore, all patients with CHC should be tested for HBV exposure prior to DAA treatment.** **In addition, HBsAg positive patients, especially those among whom have previously experienced IFN failure, should be closely monitored for HBV reactivation during DAA therapy.”**

4. The rate of SVR for the entire cohort was 87.2%, with no significant differences between the IFN-based treated, DAA-treated, and DAA treated after IFN treatment failure arms at 82.4%, 88.2%, and 92.3%, respectively.
   - It is well known that HCV pegIFN treatment patients reach low levels of SVR. How do you explain these results?

→ **Thank you for your thoughtful comment. Here is an explanation of the observed results, as you pointed out. Based on your suggestions, we have added the following to the “Discussion” section: The SVR rate in the IFN-based treatment group was generally lower than that in the DAA therapy group. However, in our study a relatively high SVR rate of 82.4% was observed in the IFN-based treatment group. Several factors could contribute to this higher rate. The study was conducted over an extended period in a single center, which may have improved the response to IFN-based treatment. The long-term follow-up and single-center cohort characteristics could have influenced the patients’ adherence to the treatment regimen, leading to a higher SVR rate. Additionally, high treatment compliance among the study participants is another crucial factor. Patients who are more compliant with their treatment regimen are more likely to achieve SVR, which could explain the higher rate observed in our study. The retrospective chart review methodology used in our study might also have contributed to the results. In summary, the higher SVR rate observed in the IFN-based treatment group in our study could be attributable to a combination of factors including cohort characteristics, treatment compliance, study design, and methodology. These factors collectively contributed to a more favorable outcome for patients treated with IFN than typically observed in other studies.**

INTRODUCTION

5. HBV/HCV coinfection is estimated to be 1%–15% worldwide [3, 4] and 1.5%–2.4% in Korea. [5, 6]
   - In abbreviated form, explain why Korea shows very low levels of HBV/HCV coinfection compared to worldwide. It is well known that Asia-Pacific countries have high levels of HBV-infected people.

→ **In the two references cited in the introduction to this paper, the prevalence of HBV and HCV co-infection in Korea was reported to be 1.5% to 2.4% [5, 6], which is lower than the rate of 1%-15% estimated worldwide [3, 4]. This discrepancy can be attributed to several factors. First, the prevalence of HBV/HCV co-infection varies widely depending on the population studied. For instance, in intravenous drug users, this prevalence can be as high as 23%, while in health promotion center controls, it is as low as 0.15%. Second, the rate of co-infection with other viruses differs among different patient groups. For example, HBsAg positivity is reported in approximately 2%-10% of anti-HCV- positive patients, and anti-HCV positivity is found in about 5%-20% of chronic hepatitis B patients. Lastly, testing methods can also influence the reported prevalence. If only HBsAg is tested or if only HCV treatment candidates with detectable HCV RNA are targeted, the prevalence of HBV co-infection in patients with hepatitis C may be underestimated, particularly in those who do not require treatment.**

**Based on your suggestions, we have added the following to the “Introduction” section: The prevalence of HBV and HCV co-infection in Korea was reported to be 1.5% to 2.4% [5, 6], which is lower than the rate of 1%-15% estimated worldwide [3, 4]. This discrepancy in HBV/HCV co-infection prevalence can be attributed to varying population demographics, differing co-infection rates with other viruses, and the influence of testing methods, which may underestimate co-infection in untreated patients.**

6. HCV superinfection in patients with chronic hepatitis B (CHB) is the most common case of HBV/HCV coinfection. [8-10]
   - is the most clinical form of HBV/HCV coinfection.

→ **In response to your suggestion, we have revised the text as follows: “HCV superinfection in patients with chronic hepatitis B (CHB) is the most clinical form of HBV/HCV co-infection. [8-10]"**

7. serum levels in patients with a previously resolved or inactive HBV infection, which is common when receiving cancer chemotherapy or taking immunosuppressive drugs after organ transplantation. [11]
   - For this affirmative sentence, the percentage of patients in immunosuppressive conditions should be added and the references cited.

→ **Based on your comments, we have made the following modifications: “HBV reactivation involves the sudden reappearance or increase in HBV DNA serum levels in patients with a previously resolved or inactive HBV infection. It is most commonly triggered by immunosuppressive therapies such as cancer chemotherapy, corticosteroids, and other immunosuppressive drugs. [11] The reactivation rate has been reported to range from 3% to 55%, particularly with rituximab in HBsAg-positive patients, [12] and is approximately 12% in non-liver solid organ transplant recipients with a history of prior HBV infection. [13]”**

8. The US Food and Drug Administration has identified reports of HBV reactivation in 29 patients (including nine patients with detectable HBV and seven hepatitis B surface antigen (HBsAg)-positive patients) who were receiving direct-acting antivirals (DAAs).

- Please, 29 patients in a cohort of how many HCV-DAA treated individuals?

→ **Thank you for your detailed feedback. Unfortunately, the exact total number of patients in the cohort was not specified in US FDA report. The specific cases of HBV reactivation identified by the FDA are as follows. The FDA identified 24 unique reports of HBV reactivation in patients receiving DAAs from November 22, 2013, to July 18, 2016. These cases included patients who were positive for hepatitis B surface antigen (HBsAg) and those with serologic evidence of resolved HBV infection. The reactivation generally occurred within 4–8 weeks of starting treatment and was associated with serious liver problems, including fulminant hepatitis, hepatic failure, and death. Two patients died and one required a liver transplant.**

9. Clinically significant HBV reactivation events are rare in the published literature on HBV reactivation in HBV/HCV coinfected patients.
   - Please, correct the sentence.

**→ Thank you for your detailed feedback. We have made the necessary corrections: “In the published literature on HBV reactivation after the treatment of hepatitis C in HBV/HCV- co-infected patients, clinically significant HBV reactivation events have been reported on rare occasions.”**

METHODS

10. All patients gave their written informed consent before starting treatment.
    - The study is retrospective and patients were initially treated in 2005. How did they sign the informed consent?

→ **HBV/HCV-co-infected patients were informed about the overall treatment process, including the procedures, medications, and potential side effects of the drugs. They were also educated on what to do if the treatment failed. Additionally, patients were informed about the importance of monitoring their HBV DNA levels and the potential risks of HBV reactivation during HCV treatment. All patients provided their informed consent for hepatitis C treatment, and the retrospective design of this study was approved by the IRB, which exempted the need for a formal consent form.**

11. Laboratory tests, including HBV serology, HBV DNA, HCV RNA, HCV genotype, complete blood counts, and liver and renal function tests were performed before treatment, after four weeks, at the end of treatment, and twelve weeks after the end of treatment (EOT), and then every six months.
    - The text should be adjusted. After treatment of HCV with DAAs, HCV-RNA is not necessary to be analyzed after each six months.

→ **Thank you for your feedback. We’ve made the appropriate modifications, as you pointed out: “Laboratory tests, including HBV serology, HBV DNA, HCV RNA, HCV genotype, complete blood counts, and liver and renal function tests were performed before treatment. HBV DNA, HCV RNA, complete blood counts, and liver and renal function tests were performed after 4 weeks of treatment, at the end of treatment, and 12 weeks after the end of treatment (EOT).”**

12. Serum AFP measurement
    - What does Serum AFP measurement mean? Alpha-fetoprotein (AFP)

**→ Thank you for your detailed feedback. We have appropriately addressed these issues.**

13. HBV-related hepatitis was defined as an alanine aminotransferase (ALT) increase of two-fold over the upper limit of normal concomitant with HBV reactivation. SVR 12 weeks after the EOT (SVR12) was used to assess the efficacy of HCV therapy.
    - A reference should be added

→ **In response to your comments, we have clarified the definition of HBV-associated hepatitis in the Methods section by adding the following references: “HBV-related hepatitis is generally defined as evidence of HBV reactivation plus an increase in alanine aminotransferase (ALT), usually more than 2-5 times the baseline value or upper limit of normal (ULN). [23, 24]”**

RESULTS

14. The age at HCV treatment was lowest in the IFN-treated group
    - This is an intriguing result. Before the DAAs regimens, due to the severe adverse events of IFN-HCV treatment, only patients with grade F3 and F4 fibrosis were treated (F3/F4). How do you explain these results? Please, correct all the paragraphs.

**→ Thank you for your thoughtful comment. Here is a revised explanation of the observed results:** **As you say, historically, interferon-based HCV treatment was generally only used in patients with more severe disease, such as advanced fibrosis, due to its serious side effects. Direct-acting antivirals, meanwhile, are well tolerated and have been shown to be effective in a wide range of patients, including those with less severe disease. The observed age difference between the IFN- and DAA-treated groups was due to the historical practice of reserving IFN-HCV treatment for patients with advanced fibrosis. This selective treatment approach resulted in younger patients being more likely to receive IFN-HCV treatment, as they were more likely to have progressed to advanced fibrosis at a younger age. Conversely, the DAA-treated group included patients who were either treated earlier in the course of their disease or who experienced treatment failure with IFN, resulting in a higher median age than in the IFN-treated group. In summary, the age difference between the IFN-treated and DAA-treated groups can be explained by the historical treatment practices and the differing tolerability profiles of these two treatment modalities.**

**We hope that this revised explanation addresses your concerns and provides a clear understanding of the observed results. Thank you again for your feedback. Based on your suggestions, we have added the following to the “Discussion” section: The age at HCV treatment was lowest in the IFN-treated group, followed by the DAA-treated group after IFN treatment failure, and then the DAA-treated group. This result is intriguing and warrants further exploration. For further clarification, it is important to note that the use of IFN-based treatments was often limited by their side effects. In patients with CHC, IFN-based therapy was generally recommended for patients with stage F2 or higher liver fibrosis and started as soon as possible for patients with advanced liver fibrosis (F3-4). In contrast, DAAs have been developed to be safer and more effective, allowing earlier treatment initiation and broader patient eligibility, including those with less severe disease. This selective treatment approach resulted in younger patients being more likely to receive IFN-HCV treatment, as they were more likely to have progressed to advanced fibrosis at a younger age. Conversely, the DAA-treated group included patients who were either treated earlier in the course of their disease or who experienced treatment failure with IFN, resulting in a higher median age than in the IFN-treated group.**

15. but there were differences in DAA regimens
    - This result was expected since for some DAA regimens, not all the drugs are pan genotypic

→ **We acknowledge your feedback and agree that the differences in DAA regimens between the IFN-treated and untreated groups are expected due to the non-pan-genotypic nature of the DAA medications used in this cohort. Accordingly, we have removed this section from the manuscript.**

16. 88.2% (15/17) in the DAA-treated group
    All other DAA regimens showed an SVR12 of 100%
    - Conflicting results, please correct, completely confused and inconsistent
    **→ Following your comments, we made the necessary changes: “The SVR12 was 82.4% (14/17) in the IFN-treated group, 88.2% (15/17) in the DAA-treated group, and 92.3% (12/13) in the DAA-treated group after IFN treatment failure. The SVR12 for different DAA treatment regimens were as follows: the daclatasvir and asunaprevir combination group achieved 87.5% (7/8), the sofosbuvir and ribavirin combination group achieved 71.4% (5/7), and all other DAA treatment regimens achieved 100% (Supplementary Figure 1), which were not significantly different.”**

17. The median interval from HCV treatment initiation to HBV reactivation was shortest in the DAA group (4.2 months), followed by 6.4 months in the DAA after IFN treatment failure group and 44.5 months in the IFN group. The interval from HCV treatment initiation to HBV reactivation was statistically significantly shorter in the DAA treatment group (4.2 months vs. 44.5 months, p < 0.001) and in the DAA after IFN treatment failure group (6.4 months vs. 44.5 months, p < 0.001) compared to the IFN group, but there was no difference between the DAA treatment group and the DAA after IFN treatment failure group.
    - Repeated information

→ **Thank you for your feedback. Here is a revised explanation of the observed results, addressing the comment about “Repeated information”: “The median interval from HCV treatment initiation to HBV reactivation was shortest in the DAA group (4.2 months), followed by the DAA after IFN treatment failure group (6.4 months) and the IFN group (44.5months). Specifically, the interval was significantly shorter in both the DAA treatment group and the DAA after IFN treatment failure group than in the IFN group (both p < 0.001). However, there was no significant difference in this regard between the DAA treatment group and the DAA after IFN treatment failure group.”**

**To clarify, we have provided a detailed comparison of the intervals from HCV treatment initiation to HBV reactivation across the different treatment groups. This comparison highlights the distinct patterns observed in each group, particularly emphasizing the shorter intervals in the DAA-treated groups than in the IFN-treated group. We have ensured that all relevant information is presented clearly and concisely to facilitate the understanding of these findings.**

18. Yes (TAF)
    - What is the meaning of TAF?

**→ Thank you for your detailed feedback. TAF is an abbreviation of “tenofovir alafenamide,” and we have correctly included this full name.**

DISCUSSION

19. The study identified HBV reactivations that occurred during or after three different HCV treatments
    - In the entire text, I suggest adding the term “regimens”, as follows: The study identified HBV reactivations that occurred during or after three different HCV treatment regimens.

**→ Thank you for your detailed feedback. We have appropriately addressed these issues.**

20. The strengths of this study are that it included a sufficiently large number of DAA-treated patients with prior IFN-based therapy...
    - The authors highlighted a sufficiently large number... Please exclude the term sufficiently, it is so evasive.

→ **Thank you for your detailed feedback. We have made the necessary corrections.**

21. The overall HBV reactivation rate in this study was slightly higher than in previous studies, the incidence of HBV reactivation in the DAA group of in this study was similar to previous studies...
    - In this paragraph the authors compared their results with others but did not hypothesize the reasons why they found similar, lower or higher results.

**→ In response to your comment, we have added the following to the discussion: “The higher overall HBV reactivation rate in this study compared to the meta-analysis findings may be due to the specific patient populations and treatment protocols used, including the presence of chronic HBV infection and previous IFN treatment failure. The similar rate of HBV reactivation in patients treated with DAAs to those in previous studies suggests that the dynamic viral patterns and baseline HBV status of patients in this cohort are representative and not heterogeneous from cohorts in previous studies.”**

22. The overall HBV reactivation rate in this study was slightly higher than in previous studies, the incidence of HBV reactivation in the DAA group of in this study was similar to previous studies.
    - The entire manuscript should be proofread and re-edited.

**→ Thank you for your detailed feedback. We will carefully review and revise the manuscript to ensure accuracy and clarity, as suggested: In this study, the rates of HBV reactivation during or after HCV treatment in HBV/HCV-co-infected patients were 31.9% in the overall cohort and 11.8%, 35.3%, and 53.8% in the IFN-based, DAA-treated, and DAA-treated patients after IFN treatment failure arms, respectively. In a previous meta-analysis, the random effects pooled overall HBV reactivation rate was 15.7% in HBsAg-positive patients receiving anti-HCV treatment. [33] The higher overall HBV reactivation rate in this study compared with the meta-analysis findings may be due to the specific patient populations and treatment protocols used, including the presence of chronic HBV infection and previous IFN treatment failure. Other studies have reported HBV reactivation rates of 36.4% in the IFN-based treatment group [16] and 30.0%-57.0% in the DAA group, [19, 27-32] and in a meta-analysis of 1060 HBV/HCV-co-infected patients, 11.9% and 21.1% in the IFN-based and DAA treatment groups, respectively. [33] The incidence of HBV reactivation in the DAA group in this study was similar to that in previous studies, [19, 27-32] and the incidence of HBV reactivation in the IFN-based group was slightly lower than that in previous studies. [16] The similar rate of HBV reactivation in patients treated with DAAs to those in previous studies suggests that the dynamic viral patterns and baseline HBV status of patients in this cohort are representative and not heterogeneous from the cohorts in previous studies.**

23. To date, no study has statistically confirmed a difference in the frequency of HBV reactivation among three diferente HCV treatments in HBV/HCV co-infected patients, but we found that HBV reactivation was more frequent in the DAA after IFN-based treatment failure group compared to the IFN-based treatment group.
    - These are the main findings of the study adding new information to the scientific literature and should be highlighted by the authors in the conclusion

→ **Your comment has been taken into account, and the conclusion is as follows: “In conclusion, HBV reactivation in HBsAg-positive CHC patients is more common and occurs earlier in those who receive DAA treatment after IFN-based treatment failure than in IFN-based treatment. Therefore, all patients with CHC should be tested for HBV exposure prior to DAA treatment. In addition, HBsAg positive patients, especially those among whom have previously experienced IFN failure, should be closely monitored for HBV reactivation during DAA therapy.”**

24. The binding of anti-HBs to secreted HBsAg to form immune complexes results in a decrease in serum anti-HBs levels, and HBV reactivation is thought to occur when the anti-HBs titer decreases [34] titers to less than 12 mIU mL-1 before and after DAA treatment [35], and high HBsAg titers before DAA treatment were associated with the risk of HBV reactivation [22].
    - In the entire manuscript there are too many long paragraphs making the text hard and boring to read.

**→ Thank you for your feedback. We will revise the manuscript to ensure that the paragraphs are shorter and more concise, making the text easier and more engaging to read. We have made the following changes to the “Discussion” section based on your comments: There is a need for further research on laboratory markers that predict HBV reactivation after HCV treatment in patients with HBV/HCV co-infection. Reactivation is known to occur when anti-HBs levels fall below 12 mIU mL^−1^ before and after DAA treatment, [38, 39] and studies have shown that high HBsAg titers before DAA treatment are associated with HBV reactivation. [26] Larger real-world studies using these markers are needed. HBV markers that predict DAA treatment-associated HBV reactivation and HBV-related hepatitis in HBV/HCV-co-infected patients require further investigation.**

CONCLUSION

25. The conclusion is not supported by the objectives, methodology and results. Furthermore, what does this study add to the field of HBV/HCV coinfected treated patients?

→ **Thank you for your comment. The study was intended to investigate the frequency and timing of HBV reactivation in patients with HBV/HCV co-infection undergoing different types of HCV treatments. The results indicate that HBV reactivation is more common and occurs earlier in patients receiving DAA treatment after IFN-based treatment failure. This study emphasizes the importance of testing all CHC patients for HBV exposure before DAA treatment and closely monitoring HBsAg-positive patients for HBV reactivation during DAA therapy. These findings contribute significantly to the understanding of the risk of HBV reactivation and provide practical guidelines for managing this risk in clinical practice, ultimately improving the care of HBV/HCV-co-infected patients.**

**Your input has been thoroughly considered, and the revised conclusion is as follows: “In conclusion, HBV reactivation in HBsAg-positive CHC patients is more common and occurs earlier in those who receive DAA treatment after IFN-based treatment failure than in IFN-based treatment. Therefore, all patients with CHC should be tested for HBV exposure prior to DAA treatment. In addition, HBsAg positive patients, especially those among whom have previously experienced IFN failure, should be closely monitored for HBV reactivation during DAA therapy.”**

**Reviewer #2:** HBV reactivation is an important issue in Hepatology, especially when using immunomodulators or immunosuppressors. It is controversial the clinical relevance of HBV reactivation in HCV/HBV coinfected patients receiving DAAs. Authors nicely try to assess this relevant topic. Congratulations for this work. I have some comments:

1. In methods section clarify the definition of HBV reactivation. You must differentiate between HBsAg pos and neg. I suggest using definitions from this reference: Mezzacappa C, Lim JK. Management of HBV reactivation: Challenges and opportunities. Clin Liver Dis (Hoboken). 2024;23(1):e0143.

→ **The analysis of this study was limited to HBsAg-positive patients. Consequently, we defined HBV reactivation according to the criteria outlined in the Methods section.**

2. In methods section clarify the definition of overt HBV infection. This term is not used for defining HBV status. I suggest using definitions from this reference: European Association for the Study of the Liver. EASL 2017 Clinical Practice Guidelines on the management of hepatitis B virus infection. J Hepatol. 2017;67(2):370-398.

**→ In this study, “overt HBV infection” refers to the HBsAg-positive status. As per your suggestion, we have replaced “overt HBV infection” with “HBsAg-positive” throughout the manuscript.**

3. In methods section clarify the definition of HBV related hepatitis. A flare is generally defined as evidence of HBV reactivation plus the ALT increase, usually more than 2–5 times of baseline value or upper limit of normal (ULN). I suggest using definitions from this reference: Huang SC, Yang HC, Kao JH. Hepatitis B reactivation: diagnosis and management. Expert Rev Gastroenterol Hepatol. 2020;14(7):565-578.

→ **Based on your comments, we have clarified the definition of HBV-related hepatitis in the Methods section as follows: “HBV-related hepatitis is generally defined as evidence of HBV reactivation plus an increase in alanine aminotransferase (ALT), usually more than 2-5 times the baseline value or upper limit of normal (ULN). [23, 24]”**

4. Please add to tables 1 and 3 all information regarding baseline HBV status in all patients.

→ **Thank you for your comment. We have added the baseline HBV status of all patients to Tables 1 and 3 as per your suggestion. And we aligned the arrangement of items in Table 1 and Table 3 for better readability.**

5. Why did you divide the cohort in 3 groups? Reactivation is related to the current treatment, not to previous treatment. I suggest using only 2 groups: DAA and IFN-based treatment. Or please explain why this is relevant.

→ **Thank you for your insightful comments on our manuscript. We understand your concern regarding the division of the cohort into three groups and appreciate your suggestion to use only two groups: DAA and IFN-based treatment. However, we would like to explain why we divided the cohort into three groups. In a clinical setting, we frequently encounter patients for whom IFN-based treatments have failed prior to initiating DAA therapy. Given the lack of existing research on the HBV reactivation rates in these specific patients, we aimed to investigate this cohort separately. By dividing the patients into three groups—those treated with DAA, those treated with IFN-based therapy, and those for whom IFN-based treatments had previously failed—we sought to comprehensively assess the risk of HBV reactivation in each subgroup. This approach allowed us to gather more detailed insights into the dynamics of HBV reactivation in different treatment contexts, particularly focusing on the unique challenges posed by patients who have experienced treatment failure with IFN-based therapies. As shown in Table 3, we observed a higher rate of HBV reactivation in the DAA treatment group than in the IFN treatment group. However, there is a dearth of existing studies specifically examining the HBV reactivation rates in patients who have previously failed IFN-based treatments before transitioning to DAA therapy. Therefore, we emphasized this aspect in our study design to address this knowledge gap. We believe that this nuanced approach is essential for understanding the broader implications of HBV reactivation in the context of HCV treatment and for developing more effective management strategies for these patients.**

6. How do you explain an 82% SVR rate in IFN-based patients?

**→ The SVR rate in the IFN-based treatment group was generally lower than that in the DAA therapy group, as you pointed out. However, in our study a relatively high SVR rate of 82.4% was observed in the IFN-based treatment group. Several factors could contribute to this higher rate. The study was conducted over an extended period in a single center, which may have improved the response to the IFN-based treatment. The long-term follow-up and single-center cohort characteristics could have influenced the patients’ adherence to the treatment regimen, leading to a higher SVR rate. Additionally, high treatment compliance among the study participants is another crucial factor. Patients who are more compliant with their treatment regimen are more likely to achieve SVR, which could explain the higher rate observed in our study. The retrospective chart review methodology used in our study might have also contributed to the results. In summary, the higher SVR rate observed in the IFN-based treatment group in our study could be attributable to a combination of factors including cohort characteristics, treatment compliance, study design, and methodology. These factors collectively contributed to a more favorable outcome for patients treated with IFN compared with that typically observed in other studies.**

**Based on your suggestions, we have added the following to the “Discussion” section: The SVR rate in the IFN-based treatment group was generally lower than that in the DAA therapy group. However, in our study a relatively high SVR rate of 82.4% was observed in the IFN-based treatment group. Several factors could contribute to this higher rate. The study was conducted over an extended period in a single center, which may have improved the response to IFN-based treatment. The long-term follow-up and single-center cohort characteristics could have influenced the patients’ adherence to the treatment regimen, leading to a higher SVR rate. Additionally, high treatment compliance among the study participants is another crucial factor. Patients who are more compliant with their treatment regimen are more likely to achieve SVR, which could explain the higher rate observed in our study. The retrospective chart review methodology used in our study might also have contributed to the results. In summary, the higher SVR rate observed in the IFN-based treatment group in our study could be attributable to a combination of factors including cohort characteristics, treatment compliance, study design, and methodology. These factors collectively contributed to a more favorable outcome for patients treated with IFN than typically observed in other studies.**

7. You wrote “The interval from HCV treatment initiation to HBV reactivation…. 44.5 Months in the IFN group”. How do you explain this? Reactivation is an event close to treatment initiation and this does not occur more than 3 years afterwards. How can you relate reactivation to IFN treatment after so many months. It may be related toother causes, or even may be spontaneous variations. Also, reactivation in IFN non responders occur at an earlier time. Please clarify this.

→ **Thank you for your insightful comments on our manuscript. We understand your concern regarding the interval from HCV treatment initiation to HBV reactivation in the IFN group. Based on the strict criterion that reactivation is an event close to the start of treatment and that it does not occur more than 3 years later, the reappearance of HBV or the 100-fold increase from baseline in the interferon-treated arm of this study at weeks 173 and 219 may not be sufficient to say that HBV reactivation is associated with interferon treatment. However, what we wanted to emphasize in this paper was the rate of HBV reactivation and the risk factors for HBV reactivation on DAA treatment. Moreover, in the real world, HBV reappearance and a 100-fold or more increase from baseline are often seen in IFN-treated patients when followed over years, HBV reactivation is more common with DAA treatment than with IFN, and DAA treatment after IFN failure is a risk factor for HBV reactivation. Therefore, we believe that our observation of delayed reactivation in the IFN group is plausible.**

8. You wrote “In univariate analysis, HBV reactivation was more frequent in the DAA-treated group than in the IFN-treated group (p = 0.037), and more specifically, the IFN-failed DAA-treated group had a higher incidence of HBV reactivation than the IFN-treated group (HR 8.750; 95% CI 1.397-54.799; p = 0.020). The incidence of HBV reactivation did not differ between the IFN-treated and DAA-treated groups or between the DAA-treated and IFN-failed DAA-treated groups”. This sentence is confusing, please clarify it.

**→ Thank you for your feedback. We understand the need for clarity in our manuscript. Here is the revised sentence: “In univariate analysis, HBV reactivation was more common in the IFN-failed DAA-treated group than in the IFN-treated group (HR 8.750; 95% CI 1.397–54.799; p = 0.020). The HBV reactivation rate did not differ between the IFN-treated and DAA-treated groups, nor between the DAA-treated and IFN-failed DAA-treated groups.”**

9. There seems to be no relation between HCC and HBV reactivation, and it is not the objective of your work. I suggest removing this section from your manuscript.

**→ We fully agree with your comment and have removed the “HCV treatment and HCC” part.**

10. Even the study goes up to June 2022, you used outdated DAAs regimes. Can you add a comment about this treatment selection?

→ **Thank you for your comment. Pan-genotypic direct-acting antiviral (DAA) agents, such as glecaprevir/pibrentasvir, became available in Korea in September 2018. Our study cohort included patients treated for hepatitis C from 2005 to 2022; therefore, many patients could not use these agents as a first-choice treatment. However, all patients in this study were treated with the most appropriate DAA regimen according to the latest guidelines of the Korean Association for the Study of the Liver at the time of their treatment. As per your comment, we have added the following information to the treatment protocol in the Materials and Methods section: “Pan-genotypic DAAs, such as glecaprevir/pibrentasvir, were available in Korea in September 2018. Therefore, the most appropriate DAA regimen for the treatment of hepatitis C was selected based on the guidelines of the Korean Association for the Study of the Liver.”**

**Reviewer #3:** Comments:
The paper entitled “Risk of HBV Reactivation among HBV/HCV Coinfected Patients Treated with Direct- Acting Antiviral Agents” by Dr. Woo group at Pusan National University Hospital, Busan, S Korea, explored the reactivation of HBV in patients with HBV/HCV infection undergoing treatment with IFN and DAA.
It is very well-analyzed clinical data over 53 months. It is interesting to see most of the HBV DNA reactivation happened in patients with high levels of HCV viremia. However, the data suggests that the reactivation was more frequent in both DAA-treated or IFN-failed DAA-treated patients than in IFN-treated patients alone. It is also evident from Table 3 that the reactivation happened in subjects with HCV genotype 1, a variant that is the most difficult to treat. The meta-analysis and results from this study suggest there is an HBV reactivation following DAA therapy, and the patients should be monitored for HBV infection for the duration of the HCV therapy.
The limitations of the study include testing the HBsAg before the treatment began, which may have provided a better clue regarding the associated risks of HBV reactivation. As the authors mentioned, it is a retrospective study, and the study design did not include the testing of HBsAg before the subjects were enrolled.
Minor comments/typos/grammar etc.:

1. There is no need to spell abbreviations everywhere except for the first time. Examples of these are HBV, HCV, NUC, and IFN.

**→ Thank you for your detailed feedback. We have corrected the text by removing the redundant abbreviations.**

2. hepatitis B envelop should be “hepatitis B envelope” on page 10.

**→ Thank you for your detailed feedback. We have modified the term to “envelope” as suggested.**

3. Pearson chi-square test should be “The Pearson chi-square test” on page 11

**→ Thank you for your detailed feedback. We have modified the term to “Pearson’s chi-square test,” as suggested.**
