# Supplementary material for: Risk of HBV reactivation in HBV/HCV-co-infected HCV-treated patients: A single-center study
Source: PLoS One. 2025 May 30;20(5):e0324019. doi: 10.1371/journal.pone.0324019 (PMC12124557; doi:10.1371/journal.pone.0324019)
Supplement: Attachment — Submitted filename: 20250402_PLOS ONE_Reviewer_comment_answer.docx [file pone.0324019.s005.docx]

Reviewer #2

Authors correctly addressed almost all reviewers’ comments.

But is still have one main concern:
It is HBV reactivation definition, especially the time frame between DAAs initiation and appearance of HBV DNA. This will impact in the study results.
The current guideline of American Association for the Study of Liver Diseases (AASLD) defines a 100-fold rise in HBV DNA level as compared to baseline, ≥1000 IU/mL in each patient with previously undetectable level or ≥10,000 IU/mL if the baseline is not available [Terrault NA, Lok ASF, McMahon BJ, et al. Update on prevention, diagnosis, and treatment of chronic hepatitis B: AASLD 2018 hepatitis B guidance. Hepatology. 2018;67(4):1560–1599].
The guideline of the Asian Pacific Association for the Study of the Liver (APASL) defines a 100-fold increase from baseline level, the new appearance of HBV DNA to a level of ≥100 or ≥20,000 IU/mL in persons without baseline level [Sarin SK, Kumar M, Lau GK, et al. Asian-Pacific clinical practice guidelines on the management of hepatitis B: a 2015 update. Hepatol Int. 2016;10(1):1–98].
The guideline of the European Association for the Study of the Liver (EASL) does not explicitly define an absolute value of a rise in HBV DNA for HBV reactivation [EASL 2017 clinical practice guidelines on the management of hepatitis B virus infection. J Hepatol. 2017;67(2):370–398].
In 2017, the FDA issued a detailed Drug Safety Communication warning about the risk of HBV reactivation (defined by the FDA as an increase greater than 1000 IU/mL in HBV DNA or detection of hepatitis B surface antigen [HBsAg] in a person who was previously negative) in some patients being treated with DAA therapy for HCV infection.
The FDA recommends that patients with a positive anti-HBc should be monitored more closely during antiviral therapy, with liver panel testing performed at least at weeks 4, 8, and 12, and after the end of treatment until sustained virologic response is achieved. [<https://www.fda.gov/drugs/drug-safety-and-availability/fda-drug-safety-communication-fda-warns-about-risk-hepatitis-b-reactivating-some-patients-treated;> Bersoff-Matcha SJ, Cao K, Jason M, Ajao A, Jones SC, Meyer T, Brinker A. Hepatitis B Virus Reactivation Associated With Direct-Acting Antiviral Therapy for Chronic Hepatitis C Virus: A Review of Cases Reported to the U.S. Food and Drug Administration Adverse Event Reporting System. Ann Intern Med. 2017;166(11):792-798.].
In almost all reports, HBV reactivation appears during DAAs and not after finishing it.
This is the same with other drugs that increase HBV reactivation risk.
Using FDA definition, the cases will be reduced, and this will be more in line with the reality of clinical practice and previous reports. If not, this report seems to overestimate HBV reactivation incidence.

I suggest the authors:

1. **To select one of the previously cited HBV reactivation definitions, especially the FDA’s since it was developed for these cases.**

→ Thank you for your insightful comments regarding our study on HBV reactivation in patients with HBV/HCV coinfection following HCV treatment. We appreciate your suggestion that our definition of HBV reactivation might be more inclusive than others, potentially leading to overdiagnosis.

In our study, HBV reactivation was defined as a 10-fold increase in HBV DNA from baseline or the detection of HBV DNA in individuals with previously undetectable levels. We acknowledge that this definition might differ from some established criteria, which often consider a 3 log IU/mL rise as indicative of reactivation. However, our definition was chosen to capture clinically significant changes in HBV DNA levels in a real-world setting.

In our study, among the 15 patients who experienced HBV reactivation, the degree of HBV DNA increase and their FIB-4 score were as follows:

| **Degree of**  **HBV DNA increase** | **DAA group** | | | **IFN group** | | |
| --- | --- | --- | --- | --- | --- | --- |
|  | Patient | LC | FIB-4 score | Patient | LC | FIB-4 score |
| **1 log** | **3 patients** | | |  | | |
|  | #5 | Yes | 15.56 |  |  |  |
|  | #6 | No | 3.74 |  |  |  |
|  | #8 | No | 2.00 |  |  |  |
| **2 log** | **7 patients** | | | **1 patient** | | |
|  | #3 | No | 2.29 | #2 | No | 10.26 |
|  | #4 | No | 10.62 |  |  |  |
|  | #7 | No | 3.79 |  |  |  |
|  | #10 | No | 3.50 |  |  |  |
|  | #11 | No | 2.46 |  |  |  |
|  | #13 | Yes | 2.74 |  |  |  |
|  | #15 | No | 5.14 |  |  |  |
| **3 log** | **3 patients** | | | **1 patient** | | |
|  | #9 | Yes | 9.01 | #1 | No | 0.69 |
|  | #12 | Yes | 2.74 |  |  |  |
|  | #14 | No | 1.43 |  |  |  |

Among the three patients with only a 1 log increase in HBV DNA, one had liver cirrhosis and the other two had advanced fibrosis (FIB-4 scores of 3.74 and 2). Despite the relatively modest increase in HBV DNA, these patients' clinical conditions suggest that even a small rise in HBV replication can be significant and warrants attention. This justification aligns with the principle that any increase in viral load, especially in patients with liver cirrhosis or advanced fibrosis, should be taken seriously.

In recent guidelines for chronic hepatitis B patients, antiviral therapy is recommended even when HBV DNA levels increase by just 1 log from undetectable levels, particularly in those with cirrhosis. This approach highlights the concern that defining HBV reactivation solely as a 3 log increase in HBV DNA may result in delayed treatment initiation based on the patient's underlying liver condition. Therefore, we propose defining HBV reactivation more broadly, such as a 1 log increase, especially for patients with advanced fibrosis, and tailoring antiviral treatment based on the patient's liver status and the occurrence of HBV-related hepatitis.

Our study aimed to observe the natural course of HBV reactivation in a real-world setting. By using a more sensitive definition, we aimed to capture early signs of reactivation that might not be caught by more stringent criteria. This approach allows for timely intervention, potentially preventing severe outcomes like liver failure or discontinuation of essential treatments.

2. **To include only patients with HBV reactivation while receiving DAAs and up to 4 to 12 weeks of finishing it. This time frame will relate DAAs with reactivation. Beyond that I think they are not related with DAAs treatment. Adapting this definition authors can submit a new report.**

→ Thank you for your valuable comments regarding our study on HBV reactivation in patients co-infected with HBV and HCV during and after DAA treatment.

In recent years, the use of various immunosuppressive agents for cancers, inflammatory bowel diseases, autoimmune disorders, and rheumatologic conditions has led to concerns about HBV reactivation in patients who had previously recovered from HBV. Similarly, HBV reactivation in HBV/HCV co-infected patients during or after DAA treatment has become a pressing concern, prompting us to conduct this study.

You suggested that we apply a criterion where HBV reactivation is defined as occurring within 12 weeks post-DAA treatment completion. In our cohort, applying the criterion suggested by the reviewer—where HBV reactivation is defined as occurring within 12 weeks after treatment completion—only nine patients met the definition. Unfortunately, we could not find guidelines that recommend defining HBV reactivation based solely on occurrence within a specific timeframe like 12 weeks post-treatment termination.

A study titled *Hepatitis B Reactivation in Patients Treated with Direct-Acting Antivirals for Hepatitis C* found that among patients receiving DAA treatment for HCV, those with HBV coinfection (35, 4.1%) and resolved HBV infection (246, 28.9%) were at risk for HBV reactivation. Specifically, HBV reactivation occurred in 10 of 29 HBsAg-positive patients (34.5%), either during DAA treatment or within 12 to 48 weeks after its completion. Additionally, HBV reactivation was observed in 2 out of 228 patients with resolved HBV infection (0.87%). These findings suggest that patients with resolved HBV infections are at a relatively low risk for HBV reactivation. Since our study also includes only HBsAg-positive patients, it is deemed reasonable to follow the progress for at least 48 weeks after completion of DAA treatment, as in the above study.

Currently, guidelines recommend maintaining prophylactic antiviral therapy for 6 to 12 months after completing treatments with drugs that pose a high risk of HBV reactivation, such as rituximab. In light of this, the suggestion to focus solely on HBV reactivation occurring within 12 weeks after DAA treatment completion seems challenging to adopt. Thus, we believe that further research is needed to clarify this aspect.

We revised the discussion section to explicitly outline the limitations of the study, including the broader definition of HBV reactivation and the extended monitoring period:

Fifth, our definition of HBV reactivation, which includes a 1 log increase in HBV DNA levels, may be more inclusive than some established criteria. While this lower threshold may lead to an overestimation of HBV reactivation rates, it aligns with the study's goal of examining the natural history of HBV reactivation in real-world clinical settings. By adopting a broader criterion, our approach also enables earlier detection of HBV reactivation, particularly in patients with advanced liver disease, which can facilitate timely intervention and potentially improve outcomes. Finally, the monitoring period in our study extended beyond the 12-week post-DAA treatment window recommended in some guidelines [18]. This broad timeframe may pose challenges when comparing results with studies that used stricter time limits, but it allowed us to capture HBV reactivation events occurring later in the course of treatment. In addition, another paper that observed HBV reactivation in CHC patients also observed follow-up for up to 48 weeks after completion of DAA treatment [43]. Current guidelines recommend maintaining prophylactic antiviral therapy for 6 to 12 months after completing treatments with high-risk drugs for HBV reactivation, suggesting that a longer monitoring period may be clinically relevant [44]. These limitations underscore the need for further research to establish standardized definitions and optimal monitoring durations for HBV reactivation in the context of DAA therapy for HCV, particularly given the potential for severe outcomes such as liver failure in some cases of HBV reactivation.
